# The Impact of Chronic Medical Conditions on the Risk of Human Metapneumovirus Hospitalizations in New Zealand Adults, 2012–2015

**DOI:** 10.1093/infdis/jiaf226

**Published:** 2025-07-16

**Authors:** Nayyereh Aminisani, Timothy Wood, Ben Waite, Ruth Seeds, Lauren Jelley, Conroy Wong, Q Sue Huang

**Affiliations:** Wallaceville Science Centre, Institute of Environmental Science and Research; Wallaceville Science Centre, Institute of Environmental Science and Research; Wallaceville Science Centre, Institute of Environmental Science and Research; Wallaceville Science Centre, Institute of Environmental Science and Research; Wallaceville Science Centre, Institute of Environmental Science and Research; Department of Respiratory Medicine, Te Whatu Ora, Health NZ Counties Manukau, Auckland; School of Medicine, University of Auckland, New Zealand; Wallaceville Science Centre, Institute of Environmental Science and Research

**Keywords:** chronic medical conditions, human metapneumovirus, incidence, acute respiratory infection, adults

## Abstract

**Background:**

Unlike influenza, information on the risk of human metapneumovirus (HMPV) infections in adults with chronic medical conditions (CMCs) is less robust.

**Methods:**

The SHIVERS project (Southern Hemisphere Influenza and Vaccine Effectiveness Research and Surveillance) performed a population-based surveillance of acute respiratory illness hospitalizations in Auckland, New Zealand, from 2012 to 2015. In this analysis, we linked these surveillance data to population-based administrative data to estimate the age- and ethnicity-adjusted risk of HMPV-associated hospitalization during the study period among adults by certain CMCs: chronic obstructive pulmonary disease, asthma, congestive heart failure, coronary artery disease, cerebrovascular accidents, diabetes mellitus, and end-stage renal disease.

**Results:**

Overall, HMPV hospitalization rates were significantly higher across all ages and ethnic groups among adults with CMCs than those without any condition. In imputed analysis, the CMC with the highest risk of HMPV hospitalization across age groups was congestive heart failure (incidence rate ratio [IRR] range, 7.0–23.0), followed by coronary artery disease (IRR range, 4.2–9.1) and chronic obstructive pulmonary disease (IRR range, 6.7–11.9) in adults aged ≥50 years. The CMC with the highest risk of HMPV hospitalization was congestive heart failure in Māori/Pacific adults and chronic obstructive pulmonary disease in non-Māori/Pacific adults. Adults with ≥2 CMCs had a higher risk than those without CMCs; the risk varied by age group and ethnicity.

**Conclusions:**

Adults with specific or ≥2 CMCs are at increased risk of HMPV hospitalizations. Age and ethnicity affect this relationship for some CMCs but not all. Such populations may benefit from future HMPV prevention strategies.

Human metapneumovirus (HMPV) is a novel respiratory virus first identified in 2001 [1]. Although HMPV infection is recognized as a cause of acute respiratory infection (ARI) in children, evidence suggests that HMPV plays an important role in respiratory infections among adults, particularly older adults [2]. HMPV-associated disease can be severe in patients who are immunocompromised, such as stem cell transplant recipients and those undergoing treatment for cancer [3, 4]. HMPV also affects older adults with chronic medical conditions (CMCs), especially older persons who are frail and have heart or lung disease [3–7]. However, previously published studies outlining the burden of HMPV may not accurately assess associated risks, since most may have had inherent selection bias by examining the prevalence of CMCs among hospitalized patients, precluding characterization of population-based HMPV risk among the general population with and without underlying conditions. Second, they focused on broad medical categories, which may limit the applicability of findings. Unlike respiratory syncytial virus (RSV) and influenza publications where population-based RSV and influenza risk among CMCs has been published [8, 9], to our knowledge, HMPV risks at the population level, by estimating the prevalence of CMCs in the catchment population data, have not been reported.

In the current study, we aimed to estimate the risk of HMPV-associated hospitalizations among adults aged 18 to 80 years with CMCs during the 2012–2015 winter seasons by using HPMV hospitalizations from an ARI surveillance platform as numerators and population-based prevalence of various CMCs as denominators. We focus on CMCs previously suggested to increase influenza/RSV-related risks [8, 9]: congestive heart failure (CHF), coronary artery disease (CAD), cerebrovascular accidents (CVAs), chronic obstructive pulmonary disease (COPD), asthma, diabetes mellitus (DM), and end-stage renal disease (ESRD).

## METHODS AND MATERIALS

### Study Population

The Southern Hemisphere Influenza and Vaccine Effectiveness Research and Surveillance (SHIVERS) project, which has been described in detail elsewhere [10], performed active population-based surveillance from 2012 to 2015 for ARI in patients admitted overnight to 4 hospitals: the Auckland City Hospital and associated Starship Children's Hospital and the Middlemore Hospital and associated Kidz First Children's Hospital. These hospitals served the central, eastern, and southern areas of Auckland, New Zealand, and were administered by the Auckland and Counties-Manukau district health boards (ADHB and CMDHB).

Study nurses evaluated all inpatient ARI cases by reviewing admission diagnoses and interviewing patients about their presenting signs and symptoms (Supplementary Materials A). They identified patients with severe ARI (SARI), defined as an ARI with fever ≥38 °C and cough within the preceding 7 days (2012) or 10 days (2013–2015) [11]. To gain a better understanding of the SARI case definition and disease burden, they additionally identified suspected ARI cases that did not meet the SARI definition (ie, ARI with cough only or measured/reported fever only, onset of cough and fever beyond 10 days). A systematic sample of patients without SARI was also interviewed and a respiratory sample collected. In 2013, 4 adult inpatients without SARI per hospital were interviewed weekly during the peak winter period (12 August–6 October). In 2014 and 2015, 12 adult patients without SARI per hospital were interviewed weekly during a broader winter period (late April–late September). This analysis included those SARI and non-SARI samples collected not only by SHIVERS study nurses but also by clinical nurses for clinical purposes (Supplementary Materials A).

### Data Collection

Study nurses obtained verbal consent from patients, completed standardized case report forms, and collected nasopharyngeal swab specimens. The case report form captured patient demographics and presenting symptoms, as well as prehospital doctor visits for the acute illness event, medication usage, influenza vaccination history, comorbidities, hospital course and outcomes, epidemiologic risk factors, and laboratory results. Data linkage for residents of ADHB or CMDHB during the study period was performed, including New Zealand Ministry of Health data collections, primary health organization records, hospital discharges (National Minimum Dataset), specialist visits (National Non-admitted Patient Collection), and birth and death records (National Maternity Collection, National Mortality Collection). Information from New Zealand Ministry of Health collections was merged to SHIVERS data via a unique identifying number (National Health Index). Using this data linkage, we were able to obtain individual-level demographic, CMC, and ARI hospitalization records for adults aged 18 to 80 years who were residing in the study area from 2012 to 2015.

### Laboratory Testing

Nasopharyngeal swabs were collected. They were tested for influenza, HMPV, RSV, parainfluenza virus 1 to 3, rhinovirus, enterovirus, and adenovirus by (1) the US Centers for Disease Control and Prevention’s real-time reverse transcription–polymerase chain reaction (RT-PCR) protocol [12, 13] at the National Influenza Centre at Institute of Environmental Science and Research, (2) the AusDiagnostics MT-PCR protocol [14] at the CMDHB laboratory, or (3) real-time RT-PCR assays at the ADHB laboratory. The performance of hospital assays from clinician-ordered specimens in the ADHB and CMDHB hospital laboratories was compared with the Centers for Disease Control and Prevention’s real-time RT-PCR as a gold standard and reported in our previous publication [5].

### CMC Case Definitions and Population-Based Prevalence Data

To define a population-based incidence, we needed to estimate the prevalence of CMCs in the catchment population to be considered as our denominator. This study was an extension of 2 previous studies, which assessed the incidence rates (IRs) of RSV and influenza and hospitalizations among adults stratified by the same CMC [8, 9]. The methods used to determine the population-based CMC prevalence of our resident populations in the study area have been outlined, and additional details are provided in the supplementary appendices in these publications [8, 9]. Details of these methods and data sources are provided in Supplementary Material B1, and variables entered into each partial least squares (PLS) model are listed in Supplementary Material B2.

Although pharmaceutical and laboratory definitions of CMCs are common, they often fail to differentiate among similar disease states. Therefore, PLS models, a type of machine learning, were developed to assess each CMC status by training the study population's health data (prescribed medications, medication class combinations, and laboratory test results) to CMC-specific hospitalization *ICD-10-CM* codes (*International Classification of Diseases, Tenth Revision, Clinical Modification*) [15]. CMC-specific health variables or health variables, or those that can differentiate closely related CMCs, were included as predictors in the PLS model. Each variable was then weighted according to its association with specific *ICD-10-CM* codes, enhancing the model's sensitivity. The optimal threshold for assigning each PLS-predicted CMC status was determined by randomly sampling 100 000 individuals and applying the Youden-modified optimality criterion [16], with expected CMC prevalences according to New Zealand CMC statistics [17]. If an individual's health data met the threshold for the model-predicted CMC status based on the model weighting, then that CMC status was assigned to the individual. Using these models, we predicted the CMC statuses for adults who did not have CMC-specific *ICD-10-CM* codes but had sufficient health data to meet the model-predicted CMC threshold. Previous studies employed similar methodologies to assign CMC status to populations [15]. However, the model was not utilized to predict CVA and ESRD status due to inaccuracies in estimating their prevalence [17]. Therefore, CVA status was assigned only by *ICD-10-CM* discharge codes for all adults. ESRD status was assigned with a combination of *ICD-10-CM* codes and laboratory results from routine medical care in New Zealand (supplementary appendices [9] and Supplementary Material B2).

### Statistical Methods

Two or more ARI hospitalizations within 14 days, including transfers to other facilities, were considered the same episode. A χ^2^ test or Fisher exact test was used to test for associations between categorical variables. The Poisson regression model was used to estimate IRs per 100 000 residents and 95% CIs. First, we assessed associations of CMCs with age, sex, prioritized ethnicity (Māori, Pacific, Asian, or European/other) [18], and socioeconomic status as estimated by the 2013 New Zealand Deprivation Index, a small-area composite measure [19]. These were used to separate the study population into socioeconomic status quintiles.

Then, we estimated the IRs by dividing the number of hospitalizations for ARIs associated with HMPV (single episodes) by the number of resident adults in the study area, categorized by their CMCs. The IR ratios (IRRs) for HMPV were calculated by dividing the IR of those individuals with CMCs by the IR of those without the corresponding CMCs (eg, COPD vs no COPD). First, we presented the IRs and IRRs for each CMC by age group (18–49, 50–64, 65–80 years) adjusted for ethnicity. Second, we estimated IRs and IRRs for each CMC by ethnicity (Māori/Pacific vs non-Māori/Pacific, including Asians and Europeans/other ethnicities), adjusted for age and socioeconomic status. We also estimated IRs and IRRs for multiple CMCs (none, 1 condition, ≥2 conditions), adjusted for ethnicity when stratified by age group and adjusted for age and socioeconomic status when stratified by ethnicity. IRs and IRRs with nonoverlapping 95% CIs and *P* values <.05 were considered significantly different. The analyses were performed in Stata version 18 (StataCorp LP).

Not all ARI hospitalizations were tested for HMPV. We verified that the not-tested patients were missing completely at random via a “missing completely at random” test [20]. We utilized the “multiple imputation by chained equations” method to address the impact of missing data. We created 30 imputed data sets for HMPV results, including age, ethnicity, socioeconomic status, the SARI case definition, week of hospitalization, all assessed CMC statuses, and specimen type (clinician ordered or study collected) as predictors of missingness [21, 22]. The imputed IRRs were not significantly different from the nonimputed IRRs for the CMCs, as shown in Supplementary Tables 3 and 4; therefore, we decided to present the imputed results, reinforcing the robustness of our findings.

## RESULTS

### Study Population

The data in Table 1 illustrate the prevalence of common CMCs based on demographic characteristics among 883 999 adult residents of Auckland aged 18 to 80 years. An overall 20% of the adult residents had at least 1 identified CMC, with the highest prevalence (44%) observed in individuals aged ≥65 years. The most prevalent CMCs in the population were asthma (12%) and DM (7%). The prevalence of CMCs varied by age, ethnicity, and socioeconomic status. About 24% of people aged ≥65 years had ≥2 condtions as opposed to <1% among those younger than 50 years.

**Table 1.** Prevalence of Estimated Chronic Medical Conditions Among Auckland Residents Hospitalized for Acute Respiratory Illness by Demographic Characteristic, New Zealand, 2012–2015

	Total		Asthma		CHF		CAD		DM		COPD^a^		CVA^a^		ESRD^a^	
	No.	%	No.	%	No.	%	No.	%	No.	%	No.	%	No.	%	No.	%
Total population	883 999	100.0	106 389	12.0	7876	0.9	28 553	3.2	64 026	7.2	31 652	11.0	4659	1.6	1777	0.6
Age, y																
18–49	597 167	100.0	61 110	10.2	617	0.1	2543	0.4	17 086	2.9	…	…	…	…	…	…
50–64	188 157	100.0	26 676	14.2	2011	1.1	10 129	5.4	26 220	13.9	15 046	8.0	1597	0.8	879	0.5
65–80	98 675	100.0	18 603	18.9	5248	5.3	15 881	16.1	20 720	21.0	16 606	16.8	3062	3.1	898	0.9
Sex																
Female	458 470	100.0	63 260	13.8	3587	0.8	9962	2.2	30 294	6.6	17 201	11.7	2082	1.4	768	0.5
Male	425 529	100.0	43 129	10.1	4289	1.0	18 591	4.4	33 732	7.9	14 451	10.3	2577	1.8	1009	0.7
Ethnic group																
Māori	80 435	100.0	14 285	17.8	1681	2.1	3048	3.8	7666	9.5	5611	29.1	493	2.6	348	1.8
Pacific	145 750	100.0	16 671	11.4	2009	1.4	5891	4.0	20 555	14.1	5494	15.3	820	2.3	786	2.2
Asian	216 145	100.0	16 853	7.8	756	0.3	5025	2.3	18 043	8.3	4127	6.7	715	1.2	256	0.4
European and others	441 669	100.0	58 580	13.3	3430	0.8	14 589	3.3	17 762	4.0	16 420	9.6	2631	1.5	387	0.2
SES^b^																
Highest quintile 1	153 589	100.0	16 900	11.0	741	0.5	4252	2.8	5950	3.9	4219	6.8	637	1.0	127	0.2
2	168 514	100.0	19 242	11.4	1077	0.6	4813	2.9	8757	5.2	4916	8.4	778	1.3	199	0.3
3	155 956	100.0	17 699	11.3	1051	0.7	4346	2.8	8779	5.6	4847	9.4	788	1.5	183	0.4
4	115 157	100.0	14 893	12.9	1120	1.0	4030	3.5	9026	7.8	4617	12.8	678	1.9	241	0.7
Lowest quintile 5	290 783	100.0	37 655	12.9	3887	1.3	11 112	3.8	31 514	10.8	13 053	16.5	1778	2.2	1027	1.3

Abbreviations: CAD, coronary artery disease; CHF, congestive heart failure; COPD, chronic obstructive pulmonary disease; CVA, cerebrovascular accident; DM, diabetes mellitus; ESRD, end-stage renal disease; SES, socioeconomic status.

^a^Modeled COPD, CVA, and ESRD data were not included for adults aged 18 to 49 years.

^b^SES is based on a small area–level measure of household deprivation derived from the national census, where quintile 1 indicates that the individual is living in a household that is in the least socioeconomic-deprived quintile.

Supplementary Table 1 shows the association of chronic conditions with demographic variables (fully adjusted model). Age was associated with an increased risk of all chronic conditions, especially among those who had CHF/CAD. When compared with those of European or other ethnicity, Maori, Pacific, and Asian ethnic groups had a higher risk of particular CMCs, even after adjusting for age. Specifically, individuals of Pacific ethnicity had a higher likelihood of having all CMCs (except asthma). Māori persons had a higher likelihood of having asthma, CHF, CAD, DM, and ESRD, and Asians showed higher DM, COPD, CVA, and ESRD. Moreover, adults from the lowest socioeconomic level were more likely to have a CMC than those of the highest level, even after adjusting for age, sex, and ethnicity (*P* < .001).

During the 2012–2015 winter seasons, there were 6911 ARI hospitalizations, including 3158 SARI cases and 3753 non-SARI cases. Of these, 3817 (55%, 3817/6911) were tested for HMPV, including 2543 SARI cases and 1274 non-SARI cases. Due to the SHIVERS study design, the testing rate was higher in the SARI hospitalizations (81%, 2543/3158) than in the non-SARI respiratory admissions (34%, 1274/3753). There were 161 (4.2%) HMPV positives: 120 (74.5%) among SARI cases and 41 (25.5%) positives among non-SARI cases (Supplementary Figure 1). There were 270 HMPV positives after dealing with the missingness by multiple imputations. Table 2 shows the number of HMPV positives among those tested hospital patients by age and ethnicity for each CMC, and it shows the imputed results.

**Table 2.** Crude and Imputed HMPV Positives and ARI Hospitalizations by Chronic Medical Conditions, Age, and Ethnicity, Auckland, New Zealand, 2012–2015

	HMPV Positives per ARI Hospitalizations															
	All		COPD^a^		Asthma		CHF		CAD		CVA^a^		DM		ESRD^a^	
	No.	%	No.	%	No.	%	No.	%	No.	%	No.	%	No.	%	No.	%
Crude																
Total	161/3817	4.2	66/1527	4.3	96/2211	4.3	34/684	5.0	54/1039	5.2	4/118	3.4	58/1074	5.4	6/142	4.2
Age, y																
18–49	50/1344	3.7	…	…	26/611	4.3	3/62	4.8	4/80	5.0	…	…	11/171	6.4	…	…
50–64	49/1114	4.4	29/601	4.8	31/679	4.6	9/193	4.7	17/336	5.1	2/36	5.6	22/411	5.4	3/77	3.9
65–80	62/1359	4.6	37/926	4.0	39/921	4.2	22/429	5.1	33/623	5.3	2/82	2.4	25/492	5.1	3/65	4.6
By ethnic group																
Māori	28/776	3.6	17/355	4.8	21/523	4.0	9/177	5.1	14/225	6.2	0/20	0.0	12/226	5.3	0/35	0.0
Pacific	67/1303	5.1	19/494	3.8	33/733	4.5	17/287	5.9	26/430	6.0	2/43	4.7	31/539	5.8	5/88	5.7
Asian	11/388	2.8	5/87	5.7	8/167	4.8	2/37	5.4	1/66	1.5	2/18	11.1	3/96	3.1	1/9	11.1
European and others	55/1350	4.1	25/591	4.2	34/788	4.3	6/183	3.3	13/318	4.1	0/37	0.0	12/213	5.6	0/10	0.0
Corrected for nontesting^b^																
Total	270/6911	3.9	125/3146	4.0	167/4225	4.0	65/1487	4.4	97/2045	4.7	10/287	3.5	98/1982	4.9	13/279	4.7
Age, y																
18–49	74/2117	3.5	…	…	37/983	3.8	5/119	4.2	7/146	4.8	…	…	16/284	5.6	…	…
50–64	79/1974	4.0	48/1153	4.2	51/1266	4.0	16/383	4.2	29/613	4.7	4/85	4.7	35/698	5.0	6/138	4.3
65–80	117/2820	4.2	77/1993	3.9	79/1976	4.0	44/985	4.5	61/1286	4.7	6/202	2.9	48/1000	4.8	7/141	5.0
Ethnic group																
Māori	47/1480	3.2	30/794	3.8	37/1084	3.4	17/428	4.0	22/465	4.7	1/53	1.9	20/453	4.4	1/79	1.3
Pacific	109/2243	4.9	39/924	4.2	58/1276	4.5	30/556	5.4	45/800	5.6	5/106	4.7	51/936	5.4	11/167	6.6
Asian	19/676	2.8	8/202	4.0	12/320	3.8	3/77	3.9	4/134	3.0	3/34	8.8	6/182	3.3	2/17	11.8
European and others	95/2512	3.8	48/1226	3.9	60/1545	3.9	16/428	3.7	26/464	5.6	2/94	2.1	21/411	5.1	0/16	0.0

Total numbers might differ slightly in each subcategory due to rounding.

Abbreviations: ARI, acute respiratory infection; CAD, coronary artery disease; CHF, congestive heart failure; COPD, chronic obstructive pulmonary disease; CVA, cerebrovascular accident; DM, diabetes mellitus; ESRD, end-stage renal disease; HMPV, human metapneumovirus.

^a^Modeled COPD, CVA, and ESRD data were not included for adults aged 18 to 49 years. Percentages are of total population by age group strata.

^b^Multiple imputation: we created 30 imputed data sets for HMPV results by the chained equations method, including age, ethnicity, socioeconomic status, the severe ARI case definition, week of hospitalization, all assessed chronic medical condition statuses, and specimen type (clinician ordered or study collected) as predictors of missingness.

Supplementary Table 2 details the proportion of HMPV-positive cases for SARI and non-SARI cases within each CMC and age group. The analysis found no significant difference in the proportion of HMPV-positive cases based on the SARI case definition among all CMCs and age groups (χ^2^ test for proportions).

### HMPV-Associated Hospitalization Rates and Rate Ratios for Each CMC, 2012–2015

The imputed IRRs were not significantly different from the crude (nonimputed) IRRs. Supplementary Tables 3 and 4 show the crude results. This section is based on the imputed findings. Tables 3 and 4 show imputed seasonal HMPV hospitalization rates and rate ratios for each CMC by age group and ethnicity.

**Table 3.** Incidence Rates per 100 000 and Incidence Rate Ratios of HMPV-Associated Hospitalizations by Age Group and Chronic Medical Condition, Auckland, New Zealand, 2012–2015, Adjusted for Ethnicity

	18–49 y		50–64 y		65–80 y	
	IR^a^ (95% CI)	IR^b^ (95% CI)	IR (95% CI)	IRR (95% CI)	IR (95% CI)	IRR (95% CI)
COPD^c^						
No	…	…	5.9 (3.4–8.3)	1 [Reference]	16.9 (10.5–23.3)	1 [Reference]
Yes	…	…	69.9 (43.3–96.5)	11.9 (6.7–21.2)	114.1 (75.2–152.9)	6.7 (4.1–11.1)
Asthma						
No	2.2 (1.4–3.1)	1 [Reference]	5.7 (3.1–8.2)	1 [Reference]	16.3 (9.9–22.7)	1 [Reference]
Yes	16.3 (10.0–22.5)	7.3 (4.2–12.6)	47.3 (30.6–64.1)	8.4 (4.7–14.9)	112.9 (75.9–149.8)	6.9 (4.2–11.4)
CHF						
No	3.7 (2.7–4.7)	1 [Reference]	10.8 (7.4–14.1)	1 [Reference]	26.1 (17.6–34.7)	1 [Reference]
Yes	87.8 (−6.4, 182.0)	23.0 (7.4–71.4)	112.4 (39.4–185.3)	10.4 (5.0–21.3)	184.1 (104.0–264.1)	7.0 (4.0–12.2)
CAD						
No	3.6 (2.6–4.6)	1 [Reference]	9.1 (6.0–12.2)	1 [Reference]	23.4 (15.4–31.3)	1 [Reference]
Yes	33.9 (.8–67.0)	9.1 (3.3–25.5)	56.8 (30.4–83.3)	6.2 (3.5–11.2)	97.7 (64.2–131.1)	4.2 (2.7–6.6)
CVA^c^						
No	…	…	12.8 (9.3–16.4)	1 [Reference]	38.2 (28.6–47.9)	1 [Reference]
Yes	…	…	42.9 (−11.3, 97.1)	3.2 (.8–12.2)	55.4 (−14.4, 125.1)	1.3 (.3–5.0)
DM						
No	3.3 (2.3–4.3)	1 [Reference]	9.6 (6.0–13.2)	1 [Reference]	32.7 (21.7–43.7)	1 [Reference]
Yes	12.7 (3.8–21.6)	3.8 (1.7–8.3)	25.8 (14.0–37.6)	2.7 (1.4–5.0)	53.5 (30.2–76.9)	1.6 (.9–2.9)
ESRD^c^						
No	…	…	12.4 (8.8–15.9)	1 [Reference]	37.2 (27.5–47.0)	1 [Reference]
Yes	…	…	93.0 (−2.2, 188.2)	7.2 (2.4–22.1)	136.1 (6.0–266.2)	3.5 (1.3–9.6)
Multiple CMCs						
0	1.8 (1.0–2.6)	1 [Reference]	3.7 (1.5–5.9)	1 [Reference]	8.0 (2.6–13.4)	1 [Reference]
1	7.9 (4.0–11.8)	4.4 (2.2–8.7)	7.0 (1.5–12.5)	1.9 (.7–5.1)	15.8 (4.2–27.5)	2.0 (.7–5.5)
≥2	35.4 (16.8–54.0)	19.5 (9.4–40.5)	57.7 (37.2–78.1)	15.8 (7.7–32.4)	104.4 (73.6–135.2)	13.1 (6.2–27.7)

Abbreviations: CAD, coronary artery disease; CHF, congestive heart failure; CMC, chronic medical condition; COPD, chronic obstructive pulmonary disease; CVA, cerebrovascular accident; DM, diabetes mellitus; ESRD, end-stage renal disease; HMPV, human metapneumovirus; IR, incidence rate; IRR, incidence rate ratio.

^a^We created 30 imputed data sets for HMPV results by the chained equations method, including age, ethnicity, socioeconomic status, the severe acute respiratory infection case definition, week of hospitalization, all assessed CMC statuses, and specimen type (clinician ordered or study collected) as predictors of missingness.

^b^IRR compares HMPV hospitalization rates between adults with and without each CMC. For multiple CMCs, IRR compares HMPV hospitalization rates between adults with 1 or more CMCs and no CMC.

^c^Modeled COPD, CVA, and ESRD data were not included for adults aged 18 to 49 years.

**Table 4.** Incidence Rates per 100 000 and Incidence Rate Ratios of HMPV-Associated Hospitalizations by Ethnicity and Chronic Medical Condition, Auckland, New Zealand, 2012–2015, Adjusted for Age and Socioeconomic Status

	Māori/Pacific^a^		Non-Māori/Pacific	
	IR^b^ (95% CI)	IRR^c^ (95% CI)	IR (95% CI)	IRR (95% CI)
COPD^d^				
No	30.0 (18.5–41.5)	1 [Reference]	4..5 (2.5–6.6)	1 [Reference]
Yes	150.8 (99.2–202.3)	5.0 (3.0–8.4)	62.7 (39.3–86.1)	13.8 (7.7–25.0)
Asthma				
No	10.4 (7.1,13.7)	1 [Reference]	2.2 (1.4–16.2)	1 [Reference]
Yes	58.5 (43.1–73.9)	5.6 (3.7–8.5)	22.1 (15.1–29.1)	9.9 (6.1–16.2)
CHF				
No	15.3 (11.6–19.1)	1 [Reference]	4.4 (3.2–5.6)	1 [Reference]
Yes	122.0 (71.5–172.6)	7.9 (4.8–12.9)	40.8 (12.6–68.9)	9.0 (4.3–18.8)
CAD				
No	13.3 (9.4–17.1)	1 [Reference]	4.2 (3.0–5.4)	1 [Reference]
Yes	81.4 (51.1–111.6)	6.3 (3.6–10.3)	16.0 (7.5–24.6)	3.8 (2.1–6.8)
DM				
No	16.3 (11.1–21.4)	1 [Reference]	4.5 (3.2–5.9)	1 [Reference]
Yes	31.2 (21.0–41.4)	1.9 (1.2–3.2)	9.6 (5.2–14.1)	2.1 (1.2–3.6)
ESRD^d^				
No	56.1 (41.5–70.7)	1 [Reference]	11.3 (7.9–14.7)	1 [Reference]
Yes	265.4 (59.6–471.2)	4.6 (2.0–10.8)	54.7 (−49.0–158.5)	4.3 (.6–33.0)
Multiple CMCs				
0	5.8 (3.0–8.7)	1 [Reference]	1.7 (1.0–2.5)	1 [Reference]
1	14.6 (7.4–21.9)	2.5 (1.2–5.1)	4.8 (2.0–7.7)	2.8 (1.3–6.0)
≥2	74.4 (52.7–96.1)	12.9 (7.0–23.7)	31.9 (19.5–44.2)	18.7 (10.2–34.4)

Abbreviations: CAD, coronary artery disease; CHF, congestive heart failure; CMC, chronic medical condition; COPD, chronic obstructive pulmonary disease; DM, diabetes mellitus; ESRD, end-stage renal disease; HMPV, human metapneumovirus; IR, incidence rate; IRR, incidence rate ratio.

^a^Māori/Pacific people were grouped because they showed a similar prevalence of CMCs (Table 2) and due to the low frequency of some conditions. They were compared with non-Māori/Pacific people, including Asians and Europeans/other ethnicities.

^b^We created 30 imputed data sets for HMPV results by the chained equations method, including age, ethnicity, socioeconomic status, the severe acute respiratory infection case definition, week of hospitalization, all assessed CMC statuses, and specimen type (clinician ordered or study collected) as predictors of missingness.

^c^IRR compares HMPV hospitalization rates between adults with and without each CMC. For multiple CMCs, IRR compares HMPV hospitalization rates between adults with 1 or more CMCs and no CMC.

^d^Modeled COPD and ESRD data were not included for adults aged 18 to 49 years.

#### Chronic Obstructive Pulmonary Disease

For adults aged ≥50 years with COPD, the rates of hospitalization due to HMPV increased significantly with age, with no statistically significant differences between the 50- to 64-year group and ≥65-year group (Table 3). Among those 50 to 64 years old, the rates of HMPV hospitalization were approximately 12 times higher for adults with COPD than those without COPD (IRR, 11.9; 95% CI, 6.7–21.2).

HMPV-associated hospitalization rates among adults with COPD varied by ethnicity, with statistically significant differences between ethnic categories (Table 4). Adults with COPD in both ethnic groups had significantly higher rates than those without COPD (IRR range, 5.0–13.8).

#### Asthma

HMPV-associated hospitalization rates increased significantly with age (Table 3). Adults with asthma had 7- to 8-times higher rates across all ages when compared with those without asthma.

HMPV-associated hospitalizations varied among adults with asthma from different ethnic backgrounds, with statistically significant differences between ethnic groups. HMPV-associated hospitalization rates in those with asthma were 5.6 to 10 times higher across ethnic groups when compared with those without asthma.

#### Congestive Heart Failure

HMPV-associated hospitalization rates among adults with CHF varied by age (Table 3). The number of 18- to 49-year-olds with CHF was low, possibly explaining a lack of significantly higher incidence in this age group. However, adults aged ≥50 years with CHF experienced 7- to 10-times higher HMPV-associated hospitalizations when compared with those without CHF.

The HMPV-associated hospitalizations varied among adults with CHF by ethnicity (Table 4). Adults with CHF across different ethnicities had 8- to 9-times higher hospitalization rates than adults without CHF (IRR range, 7.9–9.0).

#### Coronary Artery Disease

HMPV-associated hospitalizations for adults with CAD significantly increased with age (Table 3). CAD was consistently linked to higher HMPV hospitalization rates for adults of all ages, with rates 9 times higher for adults aged 18 to 49 years with CAD when compared with those without CAD.

HMPV-associated hospitalizations for adults with CAD varied by ethnicity (Table 4). HMPV-associated hospitalization rates were 6 times higher among Māori/Pacific adults with CAD than adults without CAD (IRR, 6.3; 95% CI, 3.6–10.3).

#### Cerebrovascular Accident

The number of adults with CVA was low, possibly explaining a lack of significantly higher incidence across age groups aged >50 years. For this reason, the data could not allow meaningful conclusions by age or ethnicity.

#### Diabetes Mellitus

HMPV-associated hospitalizations for adults with DM varied significantly by age (Table 3). HMPV hospitalization rates were 3 to 4 times higher for adults with DM in the 18- to 49-year and 50- to 64-year age groups when compared with those without DM but not for those in the 65- to 80-year age group.

HMPV-associated hospitalizations among adults with DM were significantly higher among people of Māori/Pacific ethnicity than European and other ethnic groups (Table 4). In both ethnic categories, those with DM were twice more likely to have HMPV-related hospitalization when compared with those without diabetes.

#### End-stage Renal Disease

HMPV hospitalization rates among adults aged ≥50 years with ESRD varied by age. However, the IRRs for HMPV hospitalization were 3.5 times higher for adults with ESRD when compared with those without ESRD in the 64- to 80-year age group. The number of 50- to 64-year-olds with ESRD was low, possibly explaining a lack of significantly higher incidence in this age group.

Adults from a Māori/Pacific background were approximately 4.6 times more likely to be hospitalized with HMPV when compared with those without ESRD. However, due to the small numbers, the results should be interpreted with caution. The figures for non-Māori/Pacific adults are inconclusive, probably due to the low number of cases.

### HMPV-Associated Hospitalization Rates and Rate Ratios for Multiple CMCs

We compared the HMPV hospitalization rates among adults with multiple CMCs (Tables 3 and 4).

We found that the rates increased in all age groups as the number of CMCs increased. HMPV hospitalization rates ranged from 7.9 to 15.8 per 100 000 for adults with 1 chronic condition, whereas for adults with ≥2 conditions, rates increased from 35.4 to 104.4 per 100 000. The HMPV IRR was the highest in adults aged <50 years with multiple CMCs when compared with those without any chronic condition (IRR, 19.5; 95% CI, 9.4–40.5).

HMPV-associated hospitalization rates were significantly higher among people of Māori/Pacific background with 1 or ≥2 conditions when compared with those from non-Māori/Pacific adults with the same categories. In both ethnic groups, the risk of HMPV hospitalization was the highest among those with ≥2 conditions (IRR range, 12.9–18.7).

## DISCUSSION

To our knowledge, this study represents the first population-based analysis of the incidence and risk of HMPV-associated hospitalizations among adults with specific CMCs from 2012 to 2015 by using population-based CMC prevalences from the source population as denominators. The risk of HMPV-associated hospitalizations was higher among adults with CHF, COPD, asthma, or CAD when compared with those without the corresponding CMC, and this risk varied depending on the specific CMC and age group. In age-stratified analyses, we found that HMPV-associated hospitalization risk was elevated in all age groups for individuals with COPD, asthma, CHF, and CAD. Yet, DM was significantly associated with higher HMPV hospitalization in young and middle-aged adults only. Results for CVA and ESRD were inconclusive due to the small number. An increased risk of being hospitalized due to HMPV was observed in adults aged 65 to 80 years with ESRD.

We showed that certain CMCs, such as CHF, CVD, and diabetes, increased the risk of HMPV-associated ARI hospitalization even in the younger group (18–49 years). Many previous studies have shown that HMPV infections mainly affect older adults with a high prevalence of cardiac and pulmonary complications, such as those of RSV infections [6, 7]. One explanation might be the unique design of the current study that enabled us to estimate the risk of HMPV hospitalization at a population level, by using the prevalence of CMCs in the catchment population as our denominator rather than only hospitalized patients as the denominators, as in the previous studies.

In our ethnicity-stratified analysis, we saw varying risks of hospitalization due to HMPV among adults with certain CMCs when compared with those without these conditions. Adults from Māori/Pacific backgrounds had a higher rate of hospitalization due to HMPV for several chronic conditions, with the highest risk for CAD and ESRD as compared with those without these conditions. Previous studies showed an increased risk of HMPV-associated hospitalizations among Māori and Pacific populations vs the non-Māori/Pacific group [5]. Research also frequently reported that these groups are at higher risk of being hospitalized for several underlying conditions [23]. However, we could not find studies to calculate the risk ratio for individual chronic conditions.

Our findings are consistent with previous hospital-based studies among adults or older adults, where CMCs were frequently observed to be significantly associated with HMPV [3, 5, 24, 25]. However, these studies could not detect the actual difference in HMPV hospitalization across different chronic conditions for several reasons. Mainly, they could be subject to Berkson bias, as the denominator was hospitalized patients: specifically, in hospital settings, there is a tendency to include older adults, who usually have a chronic condition due to their age; in addition, the possibility of being hospitalized due to HMPV is higher in this age group [26]. Another issue is that in previous studies, when it comes to chronic lung diseases, people often combine different diseases or focus only on older adults or adults with COPD [24, 25]. This makes it difficult to determine the specific impact of chronic respiratory conditions based on age group or ethnicity.

Our study has the following limitations. First, HMPV was not tested in all ARI-associated hospitalizations, and it is limited to a particular geographic region of New Zealand, which might underestimate the hospitalization rate. However, we aimed to estimate the HMPV-associated ARI hospitalization risk ratio by certain CMCs, rather than the incidence due to these conditions, which did not change after accounting for nontesting. Although the elevated risk of hospitalization due to HMPV was high for certain CMCs in adults aged 18 to 49 years, the results should be interpreted with caution due to the overall small number of such CMCs in this age group. Also, we did not consider multimorbidity in the prediction model, and each condition was weighted and modeled individually. Although we defined a variable based on combined CMCs to examine the presence of multimorbidity and the risk of HMPV-associated ARI hospitalizations in our analysis, it might not fully address the impact of multimorbidity and thus needs further assessment. Finally, our ability to assess the other CMCs, such as chronic liver conditions, disabling neurologic conditions, and immunocompromised conditions, was limited.

## CONCLUSIONS

This study provides population-based risk estimates for HMPV-associated ARI hospitalizations in adults with CMCs from 2012 to 2015. We observed a higher risk of HMPV hospitalization among adults with certain CMCs than those without these conditions. Those with 1 CMC have increased risk, those with ≥2 CMCs have even higher risk, and the risk varies by age group and ethnicity. These findings are consistent with previous studies for RSV and influenza [8, 9], indicating that certain CMCs are linked to a higher risk of hospitalizations for these viruses. We found that the HMPV hospitalization risk was increased among adults with CHF, DM, and CAD and that it was high among young adults as well. These conditions, plus COPD and asthma, were more significant among certain ethnic groups. These findings might interest policy makers for future preventive strategies and vaccine programs to target specific age and ethnic groups. The results provide insights for future research and public health initiatives in this area. Meanwhile, we appreciate that due to the small number of positives in some subgroups, the conclusions should be considered with caution. Considering the use of administrative databases, the inconsistency in testing for HMPV, the definitions of SARI and non-SARI, and the small sample size in certain comparisons, this study indicates a need for confirmation in future analyses.

## Supplementary Material

jiaf226_Supplementary_Data

## Supplementary Data

Supplementary materials are available at *The Journal of Infectious Diseases* online (http://jid.oxfordjournals.org/). Supplementary materials consist of data provided by the author that are published to benefit the reader. The posted materials are not copyedited. The contents of all supplementary data are the sole responsibility of the authors. Questions or messages regarding errors should be addressed to the author.

## Notes

***Acknowledgments.*** We greatly thank Dr Tiffany Walker and Dr Namrata Prasad for their publications with good documentation and stored files, which contributed greatly to this article. We also thank the SHIVERS investigation team: research nurses at ADHB—Kathryn Haven, Bhamita Chand, Pamela Muponisi, Debbie Aley, Claire Sherring, Miriam Rea, Judith Barry, Tracey Bushell, Julianne Brewer, and Catherine McClymont; research nurses at CMDHB—Shona Chamberlin, Reniza Ongcoy, Kirstin Davey, Emilina Jasmat, Maree Dickson, Annette Western, Olive Lai, Sheila Fowlie, Faasoa Aupa’au, and Louise Robertson; the World Health Organization National Influenza Centre, Institute of Environmental Science and Research—Pam Kawakami, Susan Walker, Robyn Madge, and Amanda des Barres; the ADHB laboratory—Fahimeh Rahnama; the CMDHB laboratory—Helen Qiao, Fifi Tse, Mahtab Zibaei, Tirzah Korrapadu, Louise Optland, and Cecilia Dela Cruz; and Labtests Laboratory in Auckland.

***Author contributions.*** Q. S. H., C. W., and T. W., R. S., designed and operationalized the cohort study. Q. S. H. and L. J. actively contributed to the testing and the metadata collection used in this analysis. N. A., T. W., and B. W. did the data analysis. N. A. wrote the first draft. All authors contributed to the interpretation of the results, revising the manuscript critically for intellectual content and giving final approval of the version to be published.

***Ethics.*** The SHIVERS study obtained ethics approval from the Northern A Health and Disability Ethics Committee (NTX/11/11/102).

***Financial support.*** The SHIVERS project is funded by the Centers for Disease Control and Prevention (grant U01IP000480). Support in kind was provided by the New Zealand Ministry of Health. This project was also conducted with support from Icosavax, a member of the AstraZeneca Group.

***Supplement sponsorship.*** This article appears as part of the supplement “Human Metapneumovirus Disease Burden in Older Adults and Implications for Adult Vaccination Strategies,” sponsored by AstraZeneca.
