# Supplementary material for: The Impact of Chronic Medical Conditions on the Risk of Human Metapneumovirus Hospitalizations in New Zealand Adults, 2012–2015
Source: J Infect Dis. 2025 Jul 16;232(Suppl 1):S59–68. doi: 10.1093/infdis/jiaf226 (PMC12265055; doi:10.1093/infdis/jiaf226)
Supplement: jiaf226_Supplementary_Data [file jiaf226_supplementary_data.docx]

**Supplementary Material A.**

**‘**Suspected acute respiratory infection’ definition

Research nurses conducted weekday assessment (and retrospective weekend day assessment) of inpatients with suspected respiratory infections or febrile illness of unknown source to establish whether each met the WHO SARI case definition or not. Prior to assessment against the SARI case definition, ‘suspected ARI’ inpatients were identified based on presentation with the following broad conditions:

- Suspected acute upper respiratory tract infection (including coryza, pharyngitis)
- Suspected croup
- Suspected pneumonia
- Exacerbations of asthma
- Exacerbations of adult chronic lung disease (including COPD, emphysema, bronchitis)
- Respiratory failure
- Febrile illness with respiratory symptoms (including shortness of breath)
- Other suspected acute respiratory infections

Broad respiratory conditions that were not considered ‘suspected ARI’ included:

- Other diseases of the upper respiratory tract e.g. allergic rhinitis
- Lung diseases due to external agents e.g. pneumonitis
- Other respiratory diseases principally affecting the interstitium e.g. Pulmonary oedema
- Suppurative and necrotic conditions of the lower respiratory tract e.g. Abscess of lung and mediastinum
- Diseases of pleura e.g. Pleural effusion not elsewhere classified, Pneumothorax
- Other diseases of the respiratory system e.g. Postprocedural respiratory disorders, not elsewhere classified

For all cases meeting the SARI case definition, an electronic record was created in the surveillance project. For adult cases that did not meet the SARI case definition criteria (i.e. non-SARI cases), electronic surveillance project records were created for a subset of patients only, with non-SARI quotas until study year 2017 and then inconsistent ward-specific non-SARI case reporting practices in subsequent years. However, non-SARI cases admitted to ICU wards were consistently reported into the surveillance project in all years across all ages.

**Supplementary Material B. Methods for estimating chronic medical condition (CMC) prevalence at the population level (1)**

**Supplementary Material B1. Data sources, Modelling**

National Minimum Dataset

The National Minimum Dataset (NMDS), a collection of ICD-10-AM hospital discharge codes obtained primarily from public hospitals throughout New Zealand, was queried for study participants between June 30, 2011 and July 1, 2016. For each individual, a NMDS CMC status was marked present if a single ICD-10AM code matching the specific CMC was found (Supplement 2) and was considered present for the entire study period**.** Those who had no codes matching the CMC were marked as absent. To reduce the likelihood a CMC status would be incorrectly marked as negative, we searched for ICD-10AM codes from June 30, 2011, to July 1, 2016, rather than limiting this search to the ARI active surveillance period (2012 to 2015).

Pharmaceutical collection

The pharmaceutical collection, a database containing data on prescription medications dispensed by community pharmacies throughout New Zealand, was used to obtain prescription medication data on study participants from June 30, 2011 to July 1, 2016. If a medication was present in the pharmaceutical collections database less than 10 times, this medication ID was not included in further analysis. As some CMC-related medications may be prescribed to treat temporary, unrelated conditions, we limited inclusion to individuals with two or more prescriptions for any CMC-related medication, thus preventing one-time users without the CMC from weakening a medication’s association in the model. We chose to mark medications as present or absent rather than counting the number of prescriptions to prevent very frequent or prolonged use of any given medication from skewing the model. Additionally, we analysed medication class combinations that help discriminate between conditions treated with similar medications (i.e., COPD and asthma) to provide specificity and context to an individual’s medication profile. Medication class combinations were marked as present if the rules for that particular combination were met (Supplement B1,2). Otherwise, the combination was marked as absent.

TestSafe laboratory database

TestSafe database is a repository of laboratory results from specimens collected from patients throughout New Zealand during routine medical care. We retrieved clinician-selected, CMC-related results from June 30, 2011 to July 1, 2016 on study participants (Appendix 2). When multiple laboratory values were available for any specific test, the result indicating the most severe status was analysed with the exception of renal function tests for which a median value was selected to attempt to capture those who had long-term impairment. Those individuals with a test result value meeting the cut-off criteria were marked as positive for that test. If they did not meet the criteria, they were marked as negative, and if there was no result for the test, they were marked as missing.

Partial Least Squares model

A predictive Partial Least Squares (PLS) model was created to define each study participant’s CMC status by training CMC-related pharmaceutical and laboratory data to NMDS CMC statuses. Relevant medications, medication group combinations, and laboratory test results for each CMC that were included in the model were determined by a three-physician consensus (Supplementary Material B2). Variables discriminating between highly related CMC in univariate logistical regression were included as predictors for training in the PLS model. Models were trained on a dataset composed of 100 individuals with at least one hospitalization between June 30, 2011 and July 1, 2016 randomly selected from age, sex, ethnicity and socioeconomic status strata as determined by the New Zealand Index of Deprivation score (2). Re-sampling and training were completed for a total of 12,000 individuals. By sampling individuals from each sociodemographic strata equally, we aimed to prevent biased predictions for individuals from uncommon sociodemographic strata. Class imbalance was further addressed with up sampling. Final models were generated using 10-fold cross validation, optimising for ROC area under the curve (3). The model was tuned over 1 through 5 terms. To adjust for the low sensitivity of NMDS CMC statuses (ICD-10AM codes), we applied a cost against false negative classification equal to the ratio of the expected CMC prevalence divided by the NMDS CMC status prevalence. By training the model to distinguish between pharmaceutical and laboratory predictors based on their association with NMDS CMC status, we more accurately captured prevalence of CMCs in the population than those captured with ICD-10AM hospitalization data alone. The optimal threshold for assigning each PLS-predicted CMC status was determined by randomly sampling 100,000 individuals from the study population and applying the Youden modified optimality criterion^2^ using expected CMC prevalence’s from available New Zealand CMC statistics (4-6).

The model was not used to predict CVA and ESRD statuses due to its inability to accurately predict the prevalence of these diseases in comparison with reported estimates. CVA status was assigned using only ICD-10 discharge codes (Supplementary Appendix 2). If a study participant had an ICD-10 code for CVA, the participant was considered to have had a CVA. ESRD status was assigned using a combination of ICD-10 discharge codes and laboratory data. To meet the ESRD status, a resident required either a qualifying ICD-10 discharge code or a qualifying laboratory value (Supplementary B2)

**Supplementary Material B2. Variables used in partial least squares model to determine CMC status of adults in the study population**

**1. Variables used in partial least squares model to determine CMC status of adults in the SHIVERS study population**

| **Variables** | **CHF** | **CAD** | **CVA** | **COPD** | **Asthma** | **DM** | **ESRD** |
| --- | --- | --- | --- | --- | --- | --- | --- |
| ICD10-AM code | I09.81  I500  I509  I11.0  I30.0  P29.0 | I200  I201  I208  I209  I210  I211  I212  I213  I214  I219  I220  I221  I228  I229  I230  I231  I232  I233  I234  I235  I236  I238  I240  I248  I249  I250  I2510  I2511  I2512  I2513  I2513  I252  I253  I254  I255  I256  I258  I259  Z951  Z955  Z958  Z959  3530306  3530307  3530400 3530401  3530500  3530501  3530906  3530907  3530908  3530909  3531000  3531001  3531002  3531003  3531004  3531005  3845619  3849700  3849701  3849702  3849703  3849704  3849705  3849706  3849707  3850000  3850001  3850002  3850003  3850004  3850300  3850301  3850302  3850303  3850304  3850500  3863700  9020100  9020101  9020102  9020103 | G460  G461  G462  G463  G464  G465  G466  G467  G468  I600  I601  I602  I603  I604  I605  I606  I607  I608  I609  I610  I611  I612  I613  I614  I615  I616  I618  I619  I630  I630  I631  I631  I632  I632  I633  I634  I635  I636  I636  I638  I639  I64  I650  I651  I652  I653  I653  I658  I658  I659  I659  I660  I661  I662  I663  I664  I668  I669  I670  I670  I672  I693  I694  I698 | J44  J41  J42  J43 | J45  J46  J82 | E10  E11  E12  E13  E14  O240  O241  O242  O243 | N18.0  Z99.2 |
| Medication | **Beta blockers:** Carvedilol Bisoprolol Metoprolol tartrate  **ACE Inhibitors:** Captopril Cilazapril Enalapril Lisinopril Perindopril Quinapril **Loop diuretics:** Furosemide Bumetanide **Potassium sparing diuretics:** Spironolactone Amiloride Metolazone **Loop w potassium sparing diuretics:** Amiloride w furosemide Amiloride w HCTZ **ACE-I w Diuretics:** Cilazapril w HCTZ Quinapril with HCTZ Captopril with HCTZ Enalaprilmaleate with HCTZ Lisinopril with HCTZ **Antiarrhythmic:** Digoxin Amiodarone | **Beta-blockers:** Atenolol Bisoprolol Carvedilol Celiprolol Labetalol Metoprolol succinate Metoprolol tartrate Nadolol Pindolol **ACE inhibitors:** Captopril Cilazapril Enalapril Lisinopril Perindopril Quinapril **ARBs:** Candesartan Losartan **Nitrates:** Glyceryl trinitrate Isosorbide mononitrate **Fibrates:** Bezafibrate Gemfibrozil **Other lipid-modifying agents:** Acipimox Nicotinic acid **Resins:** Cholestyramine Colestipol **HMG CoA Reductase Inhibitors** Atorvastatin Pravastatin Simvastatin Fluvastatin **Selective Cholesterol absorption inhibitors:** Ezetimibe Ezetimibe with simvastatin **Antiplatelet agents:** Aspirin Clopidogrel Dipyridamole Prasugrel Ticagrelor | N/a | **SAMA:** Ipratroprium Bromide,  **SABA+SAMA:** Salbutamol with Ipratroprium Bromide **LAMA:** Tiotroprium Bromide Glycopyrronium Glycopyrronium bromide Umeclidinium **IC:** Fluticasone Budesonide Beclomethasone **LABA:** Eformoterol Indacaterol Salmeterol **LAMA w LABA:** Glycopyrronium w Indacaterol Tiotropium w Olodaterol Umeclidinium w Vilanterol **IC w LABA:** Budesonide w Eformoterol Fluticasone w Vilanterol Fluticasone w Salmeterol **For exacerbation:** Salbutamol  Terbutaline  Dexamethasone Dexamethasone phosphate Methylprednisolone Methylprednisolone sodium succinate Prednisolone Prednisolone sodium phosphate Prednisone | **SABA:**  Salbutamol Terbutaline **IC:**   Fluticasone Beclomethasone Diproprionate Budesonide **LABA:** Eformoterol Fumarate Salmeterol Indacaterol **LABA w IC:** Budesonide w/ Eformoterol  Fluticasone w/ Salmeterol Fluticasone w vilanterol **Leukotriene modifier:** Montelukast **Mast cell stabilizers:** Nedocromil Sodium cromoglycate **Methylxanthines:** Theophylline Aminophylline **SAMA**: Ipratroprium Bromide **LAMA:** Tiotroprium Bromide **For exacerbation:** Salbutamol with Ipratroprium Bromide Dexamethasone Dexamethasone phosphate Methylprednisolone Methylprednisolone sodium succinate Prednisolone Prednisolone sodium phosphate Prednisone | **Insulin- Rapid-, short- and intermediate-acting preparations:** Insulin Neutral Insulin zinc suspension Insulin aspart with insulin aspart protamine Insulin isophane Insulin isophane with insulin neutral  Insulin lispro with insulin lispro protamine Insulin aspart Insulin glulisine Insulin lispro **Insulin- Long-acting preparations:** Insulin glargine **Alpha glucosidase inhibitors** Acarbose **Sulfonylureas:** Chlorpropamide Glibenclamide Gliclazide Glipizide Tolazamide Tolbutamide **Thiazolidinedione:** Rosiglitazone Pioglitazone **Other oral hypoglycaemic agents:** Metformin hydrochloride | N/a |
| Medication class combinations | BB without CCB BB and CCB ACEI withoutARB ACEI and diuretic ACEI and loop diuretic Loop diuretic and Potassium sparing diuretic Loop diuretic without Thiazide diuretic vasodilators and nitrates BB and ACEI BB and ACEI and loop diuretic CCB and ACEI BB and ARB CCB and ARB HMG CoA Reductase Inhibitors and ACEI BB and ACEI and HMG CoA Reductase Inhibitors | BB without CCB BB and CCB ACEI withoutARB vasodilators and nitrates BB and ACEI CCB and ACEI BB and ARB CCB and ARB BB and antiplatelet nitrates and antiplatelet HMG CoA Reductase Inhibitors and antiplatelet BB and ACEI and HMG CoA Reductase Inhibitors BB and HMG CoA Reductase Inhibitors and antiplatelet | N/a | SAMA and SABA SABA without SAMA SAMA without SABA SAMA and LABA SABA and LABA SABA without LABA SABA without LAMA SAMA and LAMA SABA and LAMA LABA without SAMA LABA without LAMA IC without LAMA SABA and SAMA without LABA SABA and SAMA without LAMA SABA and IC without LAMA LABA and IC LAMA and LABA SABA and IC SABA and IC and LABA SABA and LABA and IC without LAMA LAMA and IC IC and LABA and LAMA IC and LABA and LAMA Theophylline without SAMA Theophylline without LAMA Theophylline and SAMA Theophylline and LAMA corticosteroids and LAMA corticosteroids and SABA without LAMA corticosteroids and SABA without SAMA corticosteroids and SABA | SAMA and SABA SABA without SAMA SAMA without SABA SAMA and LABA SABA and LABA SABA without LABA SABA without LAMA SAMA and LAMA SABA and LAMA LABA without SAMA LABA without LAMA IC without LAMA SABA and SAMA without LABA SABA and SAMA without LAMA SABA and IC without LAMA LABA and IC LAMA and LABA SABA and IC SABA and IC and LABA SABA and LABA and IC without LAMA LAMA and IC IC and LABA and LAMA IC and LABA and LAMA Theophylline without SAMA Theophylline without LAMA Theophylline and SAMA Theophylline and LAMA corticosteroids and LAMA corticosteroids and SABA without LAMA corticosteroids and SABA without SAMA corticosteroids and SABA | Rapid insulin and long insulin sulfonylurea without Rapid insulin rapid insulin without sulfonylurea Rapid insulin and long insulin and metformin metformin and sulfonylurea sulfonylurea and long insulin | N/a |
| Laboratory tests and thresholds | BNP: 100 pg/mL or  NT-proBNP:  53 pmol/L if <50YOA,  106 pmol/L if 50-75YOA,  or  212 pmol/L if >75YOA | Troponin I or hs TroponinT: 200 ng/L | N/a | N/a | N/a | HbA1c =50mmol/mol | eGFR: <15 mL/min |

ACE-I: Angiotensin-converting enzyme inhibitor, ARB: Angiotensin receptor blocker, BB: Beta-receptor blocker, BNP: Brain natriuretic peptide, eGFR: estimated glomerular filtration rate, HbA1c: Hemoglobin A1c, HMG CoA:Beta-Hydroxy Beta-methylglutaryl-CoA, hs Troponin T: High sensitivity Troponin T, IC: inhaled corticosteroids, LABA: long-acting beta-receptor agonist, LAMA: long-acting muscarinic receptor antagonist, NT-proBNP: N-terminal pro-brain natriuretic peptide, SABA: short-acting beta-receptor agonist, SAMA: short-acting muscarinic antagonist

**2. Variables used to create case definitions for adults in the SHIVERS study population against which modelled CMC status incidences were validated.***

| **Variables** | **CHF** | **CAD** | **CVA** | **COPD** | **Asthma** | **DM** | **ESRD** |
| --- | --- | --- | --- | --- | --- | --- | --- |
| ICD10-AM code | I09.81  I500  I509  I11.0  I30.0  P29.0 | I200  I201  I208  I209  I210  I211  I212  I213  I214  I219  I220  I221  I228  I229  I230  I231  I232  I233  I234  I235  I236  I238  I240  I248  I249  I250  I2510  I2511  I2512  I2513  I2513  I252  I253  I254  I255  I256  I258  I259  Z951  Z955  Z958  Z959  3530306  3530307  3530400 3530401  3530500  3530501  3530906  3530907  3530908  3530909  3531000  3531001  3531002  3531003  3531004  3531005  3845619  3849700  3849701  3849702  3849703  3849704  3849705  3849706  3849707  3850000  3850001  3850002  3850003  3850004  3850300  3850301  3850302  3850303  3850304  3850500  3863700  9020100  9020101  9020102  9020103 | G460  G461  G462  G463  G464  G465  G466  G467  G468  I600  I601  I602  I603  I604  I605  I606  I607  I608  I609  I610  I611  I612  I613  I614  I615  I616  I618  I619  I630  I630  I631  I631  I632  I632  I633  I634  I635  I636  I636  I638  I639  I64  I650  I651  I652  I653  I653  I658  I658  I659  I659  I660  I661  I662  I663  I664  I668  I669  I670  I670  I672  I693  I694  I698 | J44  J41  J42  J43 | J45  J46  J82 | E10  E11  E12  E13  E14  O240  O241  O242  O243 | N18.0  Z99.2 |
| Medications | ≥2 Rx for one of the following:  Loop diuretics:  1544- Furosemide  1171- Bumetanide  Potassium sparing diuretics:  2176- Spironolactone  1050- Amiloride  4006- Metolazone  Loop w potassium sparing diuretics:  1051- Amiloride w furosemide | ≥2 Rx for one of the following  Nitrates:  1577 Glyceryl trinitrate  2836 Isosorbide mononitrate  Vasodilators:  3975 Nicorandil  Calcium Channel blockers:  1949- Perhexiline | N/a | ≥2 Rx for one of the following:  LAMA  SAMA | ≥2 Rx for one of the following:  LABA  IC | ≥2 Rx for one of the following  Insulin- Rapid-, short- and intermediate-acting preparations:  1648 - Insulin Neutral  1655 - Insulin zinc suspension  3982- Insulin aspart with insulin aspart protamine  1649 - Insulin isophane  6300 - Insulin isophane with insulin neutral  3982- Insulin lispro with insulin lispro protamine  3783 - Insulin aspart  3908- Insulin glulisine  1192 - Insulin lispro  Insulin- Long-acting preparations:  3857 - Insulin glargine  Alpha glucosidase inhibitors  1247 - Acarbose  Sulfonylureas:  1068 - Chlorpropamide  1567 - Glibenclamide  1568 - Gliclazide  1569 - Glipizide  2276 - Tolazamide  2277 - Tolbutamide  Thiazolidinedione:  3739 - Rosiglitazone  3800 – Pioglitazone | N/a |
| Laboratory tests and thresholds | BNP: 100 pg/mL or  NT-proBNP:  53 pmol/L if <50YOA,  106 pmol/L if 50-75YOA,  or  212 pmol/L if >75YOA | ≥2 troponin or hstroponinT:200 ng/L | N/a | N/a | N/a | ≥2 HgbA1c >=50mmol/mol | ≥2 eGFR: <15 mL/min |

***** To meet the case definition for each CMC, one must have either a single ICD-10AM code, ≥2 medication prescriptions, or ≥2 laboratory test results meeting specified threshold

BNP: Brain natriuretic peptide, eGFR: estimated glomerular filtration rate, HbA1c: Hemoglobin A1c, hs Troponin T: High sensitivity Troponin T, IC: inhaled corticosteroids, LABA: long-acting beta-receptor agonist, LAMA: long-acting muscarinic receptor antagonist, NT-proBNP: N-terminal pro-brain natriuretic peptide, SAMA: short-acting muscarinic antagonist

**References**

1. Walker TA, Waite B, Thompson MG, McArthur C, Wong C, Baker MG, et al. Risk of Severe Influenza Among Adults With Chronic Medical Conditions. The Journal of infectious diseases. 2020;221(2):183-90.

2. Atkinson J, Salmond C, Crampton P. NZDep2013 index of deprivation. New Zealand, . 2014.

3. Perkins NJ, Schisterman EF. The inconsistency of "optimal" cutpoints obtained using two criteria based on the receiver operating characteristic curve. American journal of epidemiology. 2006;163(7):670-5.

4. Telfar Barnard L BM, Pierse N, et al. . The impact of respiratory disease in New Zealand: 2014 update Wellington: The Asthma Foundation; 2015 [Available from: <https://www.asthmafoundation.org.nz/research/the-impact-of-respiratory-disease-in-new-zealand-2014-update>.

5. Annual Update of Key Results 2015/16: New Zealand Health Survey. . Wellington: Ministry of Health.; 2016.

6. Managing Chronic Kidney Disease in Primary Care: National Consensus Statement. Wellington: Ministry of Health. . Wellington: Ministry of Health; 2015.

**Supplementary Figure S1. Southern Hemisphere Influenza Vaccine Effectiveness Research and Surveillance (SHIVERS) study enrollment status for adults aged ≥20 y hospitalized with acute respiratory infection (ARI), stratified by severe acute respiratory infection (SARI) and testing status for human metapneumovirus (HMPV), Auckland, New Zealand, 2012–2015.**

883999 study population

120 HMPV +

(Clinician-ordered HMPV+: 9)

41 HMPV+ (Clinician-ordered HMPV+: 28)

3158

SARI** cases

3753

Non-SARI*** cases

2543 tested for hMPV

(Clinician-ordered tests: 305)

1274 tested for hMPV

(Clinician-ordered tests:775)

Not tested

Non-SARI cases

Not-tested

SARI cases

2012–2015 winter seasons, 6911

ARI* hospitalizations

* ARI, acute respiratory infection

** SARI, severe ARI, defined as ARI with a history of fever or measured fever ≥ 38^◦^C and cough in the preceding 10 days with onset in the preceding

*** Non-SARI non-SARI respiratory patients with cough and/or measured or reported fever but not both, within 10 days

| **Supplementary Table S1: Associations of chronic medical conditions with demographic factors in the source population** **during 2012-2015, Auckland, New Zealand** | | | | | | | | | | | | | | | | | | | | | | | |
| --- | --- | --- | --- | --- | --- | --- | --- | --- | --- | --- | --- | --- | --- | --- | --- | --- | --- | --- | --- | --- | --- | --- | --- |
| **Characteristics** | **Asthma** | | **Congestive Heart Failure (CHF)** | | | **Coronary Artery Disease (CAD)** | | | | **Diabetes Mellitus**  **(DM)** | | | | **Chronic Obstructive Pulmonary Disease (COPD)*** | | | **Cerebrovascular Accident (CVA)*** | | | **End-stage renal disease (ESRD)*** | | |  |
|  | **IRR (95%CI)** | | **IRR (95%CI)** | | | **IRR (95%CI)** | | | | **IRR (95%CI)** | | | | **IRR (95%CI)** | | | **IRR (95%CI)** | | | **IRR (95%CI)** | | |  |
| **Age group (years)** |  | |  | | |  | | | |  | | | |  | | |  | | |  | | |  |
| 18-49 | Ref | | Ref | | | Ref | | | | Ref | | | |  | | |  | | |  | | |  |
| 50-64 | 1.23 (1.21, 1.25) | | 10.55 (9.64, 11.54) | | | 11.93 (11.43, 12.46) | | | | 5.06 (4.97, 5.15) | | | | Ref | | | Ref | | | Ref | | |  |
| 65-80 | 1.69 (1.67, 1.72) | | 60.82 (55.99, 66.05) | | | 39.01 (37.43, 40.66) | | | | 8.86 (8.70, 9.03) | | | | 2.32 (2.28, 2.37) | | | 3.94 (3.71, 4.18) | | | 2.41 (2.20, 2.64) | | |  |
| **Sex** |  | |  | | |  | | | |  | | | |  | | |  | | |  | | |  |
| Female | Ref | | Ref | | | Ref | | | | Ref | | | | Ref | | | Ref | | | Ref | | |  |
| Male | 0.75 (0.74, 0.76) | | 1.32 (1.26, 1.38) | | | 2.02 (1.97, 2.07) | | | | 1.23 (1.21, 1.24) | | | | 0.91 (0.89, 0.93) | | | 1.33 (1.26, 1.41) | | | 1.46 (1.33, 1.60) | | |  |
| **Ethnicity** |  | |  | | |  | | | |  | | | |  | | |  | | |  | | |  |
| Māori | 1.35 (1.32, 1.37) | | 3.99 (3.75, 4.24) | | | 1.80 (1.73, 1.87) | | | | 2.74 (2.67, 2.82) | | | | 0.71 (0.69, 0.73) | | | 0.81 (0.74, 0.88) | | | 7.23 (6.18, 8.45) | | |  |
| Pacific | 0.81 (0.79, 0.82) | | 2.10 (1.98, 2.24) | | | 1.60 (1.55, 1.66) | | | | 3.56 (3.49, 3.64) | | | | 1.27 (1.23, 1.31) | | | 1.26 (1.15, 1.37) | | | 7.55 (6.56, 8.70) | | |  |
| Asian | 0.61 (0.60, 0.62) | | 0.63 (0.59, 0.69) | | | 0.99 (0.96, 1.02) | | | | 2.50 (2.45, 2.55) | | | | 2.75 (2.67, 2.83) | | | 1.67 (1.51, 1.85) | | | 1.88 (1.61, 2.21) | | |  |
| European and others | Ref | | Ref | | | Ref | | | | Ref | | | | Ref | | | Ref | | | Ref | | |  |
| **SES^**^** |  | |  | | |  | | | |  | | | |  | | |  | | |  | | |  |
| Highest quintile | Ref | | Ref | | | Ref | | | | Ref | | | | Ref | | | Ref | | | Ref | | |  |
| 2 | 1.11 (1.09, 1.13) | | 1.50 (1.37, 1.65) | | | 1.18 (1.13, 1.23) | | | | 1.32 (1.28, 1.36) | | | | 1.25 (1.20, 1.30) | | | 1.33 (1.20, 1.47) | | | 1.38 (1.10, 1.73) | | |  |
| 3 | 1.14 (1.12, 1.16) | | 1.57 (1.43, 1.72) | | | 1.18 (1.13, 1.22) | | | | 1.42 (1.38, 1.47) | | | | 1.36 (1.31, 1.41) | | | 1.49 (1.35, 1.66) | | | 1.32 (1.05, 1.66) | | |  |
| 4 | 1.29 (1.26, 1.31) | | 2.09 (1.90, 2.29) | | | 1.47 (1.41, 1.53) | | | | 1.75 (1.70, 1.81) | | | | 1.73 (1.67, 1.80) | | | 1.79 (1.60, 2.00) | | | 1.82 (1.45, 2.27) | | |  |
| Lowest quintile | 1.32 (1.29, 1.34) | | 2.53 (2.33, 2.76) | | | 1.62 (1.56, 1.68) | | | | 2.09 (2.03, 2.15) | | | | 1.96 (1.89, 2.03) | | | 2.02 (1.83, 2.23) | | | 2.15 (1.75, 2.64) | | |  |
| *It was calculated for people ≥50 years | | |  | |  |  | |  |  |  | |  |  |  | |  |  | |  |  | |  |  |
| ^**^ SES was based on a small area–level measure of household deprivation derived from the national census, where 1 indicates the individual is living in a household that is in the least socioeconomic-deprived quintile. | | | | | | | | | | | | | | | | | | | | | | | |

| **Supplementary Table S2. Proportion positive for Human metapneumovirus (HMPV) by SARI case definition for each chronic medical condition and age group among hospital patients** | | | | | | |
| --- | --- | --- | --- | --- | --- | --- |
|  | **18-49 years** | | **50-64 years** | | **65-80 years** | |
|  | **SARI** | **Non-SARI** | **SARI** | **Non-SARI** | **SARI** | **Non-SARI** |
| **Chronic Obstructive Pulmonary Disease (COPD)*** |  |  |  |  |  |  |
| HMPV positive/Tested hospitalisation |  |  | 21/372 | 8/229 | 25/548 | 12/378 |
| % |  |  | 5.7 | 3.4 | 4.6 | 3.2 |
| *P-value* |  |  | *0.232* | | *0.289* | |
| **Asthma** |  |  |  |  |  |  |
| HMPV positive/Tested hospitalisations | 19/403 | 7/208 | 24/430 | 7/249 | 26/543 | 13/378 |
| % | 4.7 | 3.4 | 5.9 | 2.8 | 4.8 | 3.4 |
| *P-value* | *0.434* | | *0.096* | | *0.317* | |
| **Congestive Heart Failure (CHF)** |  |  |  |  |  |  |
| HMPV positive/Tested hospitalisations | 3/40 | 0/22 | 6/123 | 3/70 | 13/264 | 9/165 |
| % | 7.5 | 0 | 4.9 | 4.3 | 4.9 | 5.5 |
| *P-value* | *0.546* | | *>0.99* | | *0.809* | |
| **Coronary Artery Disease (CAD)** |  |  |  |  |  |  |
| HMPV positive/Tested hospitalisations | 4/60 | 0/20 | 12/234 | 5/102 | 22/392 | 11/231 |
| % | 6.7 | 0 | 5.1 | 4.9 | 5.6 | 4.8 |
| *P-value* | *0.567* | | *0.931* | | *0.647* | |
| **Cerebrovascular Accident (CVA)*** |  |  |  |  |  |  |
| HMPV positive/Tested hospitalisations |  |  | 1/28 | 1/8 | 2/54 | 0/28 |
| % |  |  | 3.6 | 12.5 | 3.7 | 0 |
| *P-value* |  |  | *0.4* | | *0.545* | |
| **Diabetes Mellitus (DM)** |  |  |  |  |  |  |
| HMPV positive/Tested hospitalisations | 10/128 | 1/43 | 18/286 | 4/125 | 14/313 | 11/179 |
| % | 7.8 | 2.3 | 6.3 | 3.2 | 4.5 | 6.2 |
| *P-value* | *0.294* | | *0.241* | | *0.523* | |
| **End-stage renal disease* (ESRD)** |  |  |  |  |  |  |
| HMPV positive/Tested hospitalisations |  |  | 3/57 | 0/20 | 2/39 | 1/26 |
| % |  |  | 5.3 | 0 | 5.1 | 3.9 |
| *P-value* |  |  | *0.564* | | *>0.99* | |

*It was calculated for people ≥50 years, if the numbers were <5, the Fisher exact test was performed

| **Supplementary Table S3: Crude Incidence Rates per 100,000 and Incidence Rate Ratios of Human metapneumovirus (HMPV)-Associated Hospitalisations by Age Group and Chronic Medical Condition, Auckland New Zealand, 2012–2015, Adjusted for Ethnicity** | | | | | | |
| --- | --- | --- | --- | --- | --- | --- |
|  | **18-49 years** | | **50-64 years** | | **65-80 years** | |
|  | **IR* (95% CI)** | **IRR** (95% CI)** | **IR (95% CI)** | **IRR (95% CI)** | **IR (95% CI)** | **IRR (95% CI)** |
| **Chronic Obstructive Pulmonary Disease (COPD)***** |  |  |  |  |  |  |
| No |  |  | 3.4 (1.9, 4.9) | Ref | 9.8 (5.9, 13.7) | Ref |
| Yes |  |  | 39.6 (23.1, 56.1) | 11.6 (6.2, 21.8) | 48.1 (30.8, 65.4) | 4.9 (2.8, 8.6) |
| **Asthma** |  |  |  |  |  |  |
| No | 1.4 (0.9, 2.0) | Ref | 3.3 (1.8, 4.9) | Ref | 9.1 (5.4, 12.8) | Ref |
| Yes | 11.6 (7.0, 16.1) | 8.0 (4.6, 14.2) | 26.5 (16.6, 36.5) | 8.0 (4.4, 14.6) | 49.5 (33.5, 65.5) | 5.4 (3.2, 9.2) |
| **Congestive Heart Failure (CHF)** |  |  |  |  |  |  |
| No | 2.5 (1.8, 3.2) | Ref | 6.2 (4.2, 8.2) | Ref | 13.1 (9.1, 17.2) | Ref |
| Yes | 47.1 (-6.5, 100.7) | 18.8 (5.8, 60.7) | 58.6 (15.4, 101.8) | 9.4 (4.1, 21.5) | 82.5 (44.2, 120.8) | 6.3 (3.5, 11.1) |
| **Coronary Artery Disease (CAD)** |  |  |  |  |  |  |
| No | 2.5 (1.8, 3.2) | Ref | 5.3 (3.5, 7.1) | Ref | 11.0 (7.1, 15.0) | Ref |
| Yes | 17.4 (0.1, 34.7) | 7.1 (2.5, 19.8) | 30.9 (14.4, 47.4) | 5.8 (3.0, 11.2) | 48.6 (31.9, 65.3) | 4.4 (2.7, 7.2) |
| **Cerebrovascular Accident (CVA)***** |  |  |  |  |  |  |
| No |  |  | 7.3 (5.1, 9.4) | Ref | 18.8 (14.1, 23.6) | Ref |
| Yes |  |  | 23.9 (-9.7, 57.4) | 3.3 (0.8, 13.9) | 16.1 (-6.3, 38.5) | 0.9 (0.2, 3.5) |
| **Diabetes Mellitus (DM)** |  |  |  |  |  |  |
| No | 2.2 (1.5, 2.9) | Ref | 5.2 (3.2, 7.3) | Ref | 16.0 (10.7, 21.3) | Ref |
| Yes | 8.3 (2.3, 14.3) | 3.7 (1.6, 8.4) | 15.4 (7.8, 23.0) | 2.9 (1.5, 5.7) | 25.0 (14.4, 35.6) | 1.6 (0.9, 2.8) |
| **End-stage renal disease (ESRD)***** |  |  |  |  |  |  |
| No |  |  | 7.1 (5.0, 9.2) | Ref | 18.1 (13.5, 22.7) | Ref |
| Yes |  |  | 44.0 (-7.9, 96.0) | 6.2 (1.8, 21.1) | 53.5 (-7.2, 114.3) | 3.0 (0.9, 9.5) |
| **Multiple Chronic Medical Conditions** |  |  |  |  |  |  |
| None | 1.2 (0.62, 1.7) | Ref | 2.6 (1.0, 4.3) | Ref | 4.9 (1.2, 8.5) | Ref |
| One condition | 6.0 (2.9, 9.0) | 5.2 (2.6, 10.4) | 4.9 (1.0,8.9) | 1.9 (0.7, 5.3) | 10.2 (2.6, 17.8) | 2.1 (0.8, 6.1) |
| Two or more conditions | 22.2 (9.7, 34.8) | 19.2 (9.1, 40.6) | 34.8 (21.2, 48.4) | 13.3 (6.1, 29.2) | 53.8 (38.0, 69.5) | 11.1 (4.9, 25.0) |

* IR, incidence rate; IRR, incidence rate ratio.

** IRR compares HMPV hospitalisation rates between adults with vs without each chronic medical condition. For multiple CMs, IRR compares HMPV hospitalisation rates between adults with 1 or more CMCs and no CMC.

*** Modelled COPD and ESRD data were not included for adults aged 18–49.

| **Supplementary Table S4: Crude Incidence Rates per 100,000 and Incidence Rate Ratios of Human metapneumovirus (HMPV)-Associated Hospitalisations by Ethnicity and Chronic Medical Condition, Auckland New Zealand, 2012–2015** | | | | |
| --- | --- | --- | --- | --- |
|  | **Māori/Pacific¥** | | **Non-Māori/Pacific** | |
|  | **IR* (95% CI)** | **IRR** (95% CI)** | **IR (95% CI)** | **IRR (95% CI)** |
| **Chronic Obstructive Pulmonary Disease (COPD)***** |  |  |  |  |
| No | 19.9 (12.2, 27.5) | Ref | 2.6 (1.4, 3.9) | Ref |
| Yes | 83.1 (52.9, 113.3) | 4.2 (2.5, 7.1) | 36.4 (22.1, 50.8) | 13.9 (7.4, 26.1) |
| **Asthma** |  |  |  |  |
| No | 6.8 (4.7, 8.9) | Ref | 1.3 (0.77, 1.8) | Ref |
| Yes | 35.1 (25.1, 45.1) | 5.1 (3.4, 7.9) | 14.4 (9.6, 19.2) | 10.5 (6.3, 17.3) |
| **Congestive Heart Failure (CHF)** |  |  |  |  |
| No | 9.6 (7.3, 11.9) | Ref | 2.7 (2.0, 3.4) (3.0, 5.4) | Ref |
| Yes | 79.2 (43.6, 114.9) | 8.3 (4.9, 13.9) | 19.9 (4.3, 35.5) | 7.4 (3.3, 17.0) |
| **Coronary Artery Disease (CAD)** |  |  |  |  |
| No | 8.1 (6.0, 10.3) | Ref | 2.6 (1.9, 3.3) | Ref |
| Yes | 53.9 (33.7, 74.1) | 6.7 (4.2, 10.6) | 8.3 (3.3, 13.2) | 3.2 (1.7, 6.3) |
| **Diabetes Mellitus (DM)** |  |  |  |  |
| No | 9.7 (6.9, 12.4) | Ref | 2.6 (1.9, 3.4) | Ref |
| Yes | 19.8 (12.9, 26.7) | 2.0 (1.3, 3.3) | 5.8 (2.7, 8.9) | 2.2 (1.9, 4.0) |
| **End-stage renal disease (ESRD)***** |  |  |  |  |
| No | 3.3 (2.4, 4.2) | Ref | 6.2 (4.4, 8.0) | ref |
| Yes | 131.0 (16.0, 246.0) | 3.9 (1.6, 9.9) | 35.9 (-36.1, 107.8) | 5.8 (0.76, 44.1) |
| **Multiple Chronic Medical Conditions** |  |  |  |  |
| None | 3.9 (2.0, 5.8) | Ref | 1.0 (0.54,1.5) | Ref |
| One condition | 11.2 (5.5, 17.0) | 2.9 (1.4, 6.0) | 3.4 (1.4, 5.4) | 3.3 (1.5, 7.1) |
| Two or more conditions | 44.3 (30.1, 58.5) | 11.4 (6.1, 21.2) | 19.6 (10.9, 28.4) | 19.1 (9.7, 37.8) |
| * *** Modelled COPD and ESRD data were not included for adults aged 18–49.  ¥ Māori/Pacific people were grouped together, because they showed similar prevalence of CMCs (Table 2) and due to low frequency of some conditions. They were compared with non-Māori/Pacific (including Europeans, Asians & other ethnicities). | | | | |
| * IR, incidence rate; IRR, incidence rate ratio adjusted for age and socioeconomic status. | | | | |

** IRR compares HMPV hospitalisation rates between adults with vs without each chronic medical condition. For multiple CMs, IRR compares HMPV hospitalisation rates between adults with 1 or more CMCs and no CMC.

*** Modelled COPD and ESRD data were not included for adults aged 18–49.
